# Multiple Roles of the RUNX Gene Family in Hepatocellular Carcinoma and Their Potential Clinical Implications

**DOI:** 10.3390/cells12182303

**Published:** 2023-09-19

**Authors:** Milena Krajnović, Bojana Kožik, Ana Božović, Snežana Jovanović-Ćupić

**Affiliations:** Laboratory for Radiobiology and Molecular Genetics, Vinča Institute of Nuclear Sciences, National Institute of the Republic of Serbia, University of Belgrade, Mike Petrovića Alasa 12-14, Vinča, 11351 Belgrade, Serbia; mdragic@vin.bg.ac.rs (M.K.); anabozovic@vin.bg.ac.rs (A.B.); cupic@vin.bg.ac.rs (S.J.-Ć.)

**Keywords:** RUNX, hepatocellular carcinoma, oncogenes, tumour suppressors, biomarkers

## Abstract

Hepatocellular carcinoma (HCC) is one of the most frequent cancers in humans, characterised by a high resistance to conventional chemotherapy, late diagnosis, and a high mortality rate. It is necessary to elucidate the molecular mechanisms involved in hepatocarcinogenesis to improve diagnosis and treatment outcomes. The Runt-related (RUNX) family of transcription factors (RUNX1, RUNX2, and RUNX3) participates in cardinal biological processes and plays paramount roles in the pathogenesis of numerous human malignancies. Their role is often controversial as they can act as oncogenes or tumour suppressors and depends on cellular context. Evidence shows that deregulated *RUNX* genes may be involved in hepatocarcinogenesis from the earliest to the latest stages. In this review, we summarise the topical evidence on the roles of *RUNX* gene family members in HCC. We discuss their possible application as non-invasive molecular markers for early diagnosis, prognosis, and development of novel treatment strategies in HCC patients.

## 1. Introduction

Hepatocellular carcinoma is a primary liver cancer and one of the leading causes of mortality with wide geographic variation [1]. Any factor that leads to chronic liver injury and cirrhosis can be considered an oncogenic agent. Prevalent HCC risk factors are Hepatitis viruses B and C infection, excessive alcohol intake, nonalcoholic steatohepatitis (NASH), and aflatoxin B1 exposure [2,3].

The major problem with the HCC treatment is the diagnosis is usually made in progressed disease stadiums when conventional systemic chemotherapy is ineffective [4]. To overcome this problem, researchers are developing molecularly targeted treatments that represent a more promising approach for advanced HCC. Several such drugs are clinically available, but their efficacy is limited [5]. Therefore, elucidating the molecular processes at the basis of hepatocarcinogenesis is critical for improving therapy outcomes and diagnosis.

The family of Runt-related (*RUNX*) genes (*RUNX1*, *RUNX2*, and *RUNX3*) is crucial for the different tumour types’ development and progression. RUNX proteins are transcription factors that behave in opposing ways, promoting or suppressing tumourigenesis [6,7,8]. However, the exact mechanisms of their deregulation in HCC, especially for RUNX1 and RUNX2, have not yet been sufficiently investigated.

### 1.1. RUNX Genes’ and Proteins’ Structure

**Figure 1:**
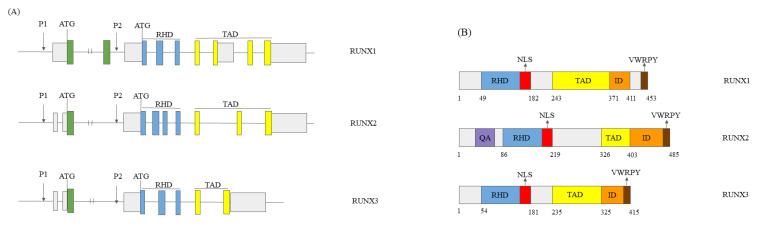
The structures of RUNX1, RUNX2, and RUNX3 genes and proteins. (**A**) *RUNX1*, *RUNX2*, and *RUNX3* genes’ structure. Rectangles—exons; lines—introns; ATG—start codon; P1 and P2—promoters, RHD—Runt homology domain; and TAD—transactivation domain. Grey rectangles—untranslated regions. (**B**) RUNX1, RUNX2, and RUNX3 proteins’ structure. Rectangles—protein domains. Numbers—amino acids’ numbers. NLS—nuclear localisation signal; QA—the glutamine/alanine-rich signal, RUNX2 specific; ID—inhibitory domain; and VWRPY—Groucho/TLE binding site. The figure is a not-to-scale drawing. We created the figure under CC BY NC, based on Yi et al., 2022 [10].

Human *RUNX* genes participate in neuro-, blood, and bone development [7,8]. The role in developmental processes implies tight control of *RUNX* genes at transcriptional and posttranscriptional levels. *RUNX* genes’ chromosome location differs depending on the species, and human *RUNX1, RUNX2,* and *RUNX3* genes’ locations are on chromosomes 21, 6, and 1, respectively. Two alternative promoters of *RUNX* genes, P1 and P2, generate two dominant isoforms with differing 5′-untranslated regions (5′-UTRs) (Figure 1A). These transcripts give two polypeptides with different N-terminal sequences, distal and proximal [9]. The diversity of RUNX transcripts is the result of alternative splicing. RUNX1 and RUNX2 have nine exons and twelve isoforms, whereas RUNX3 has six exons and two isoforms [7,10]. RUNX proteins’ molecular weight is 44, 50, and 57 KDa [7]. 

RUNX proteins received their name from the Runt homology domain (RHD) on the protein N-terminus, which has approximately 90% sequence homology in all three proteins [6]. The RHD domain binds to the target genes’ DNA through the consensus sequence of seven nucleotides, ‘PyGPyGGTPy’. Nuclear localisation and interaction with the other proteins are also functions of the RHD domain [10]. The RUNX proteins’ C-terminus encompasses the transactivation domain (TAD) and inhibitory domain (ID), with the consensus sequence of five amino acids, valine–tryptophan–arginine–proline–tyrosine (VWRPY). This sequence recruits Groucho/Transducin-like enhancer protein (TLE) corepressors [7,10,11]. TLE corepressors further control several target genes’ transcription. The TAD domain is the place of RUNX proteins’ interactions with the other regulatory proteins and control of their transcriptional activity [12]. In complex with the coregulators, RUNX proteins influence various cell processes [10] (Figure 1B).

Posttranscriptional modifications of RUNX affect their overexpression or loss of function, indicating RUNX’s dual role [13]. The RUNX proteins also undergo post-translational modifications [8], further influencing cell cycle regulation and response to external stimuli [6].

### 1.2. The Role of the RUNX Genes in Normal Development

*RUNX* genes engage in normal cell development and differentiation processes, playing a tissue-specific role [14]. *RUNX1* is crucial for cell growth and differentiation of immune cells, epithelial stem and epithelial cells, and neurodevelopment [11,15]. *RUNX1* acts in the development of hematopoietic cells in vertebrates [13,16]. Consequently, the chromosomal rearrangements and gene mutations involving *RUNX1* lead to various leukaemia types [13]. RUNX2 is a factor in bone generation [11]. *RUNX2* knockout mice lack osteoblast differentiation, leading to osteoporosis [17]. *RUNX3* is essential for embryogenesis, and *RUNX3* knockout mice die quickly [18]. Additional studies have shown that RUNX3 participated in nervous and gastrointestinal system development, bone, and immune cells [11,19]. We used multi-omics datasets from the atlas of the healthy human liver [20] to examine RUNX-specific tissue expression in different liver cell compartments. According to the Liver Cell Atlas datasets, the overall RUNX1, RUNX2, and RUNX3 expression levels were 14.9%, 7.6%, and 25.1%, respectively [21].

### 1.3. The Role of RUNX Genes in Cancer

*RUNX* genes’ mutations and abnormal expression lead to various cancer types. RUNX genes can hinder or activate tumourigenesis [12]. A recent study by Pan and colleagues [22] suggests that *RUNX* genes’ aberrant expression causes disparate cancer types and influences disease prognosis.

*RUNX1* plays a cardinal role in hematopoiesis and, consequently, in haematological tumours. The researchers documented mutations of the *RUNX1* gene in acute myeloid leukaemia (AML) [23], acute lymphoid leukaemia (ALL), and familial platelet disorder with a predisposition to acute myeloid leukaemia (FPD/AML). These mutations either influence or do not influence the binding ability of RUNX1 to the other target genes, with the more or less changed function, depending on the mutated region (DBD, TAD, or nuclear localisation domain) (as reviewed in [24]). The prevalent mutations are point mutations in the DBD of RUNX1 in AML or FPD germ line mutations [25]. Frameshift mutations and stop codons that remove TAD are found in AML [26,27] or FPD [25] and are often associated with loss of function. Chromosomal rearrangements, like translocations, t (8; 21), t (3; 21), and t (12; 21), which form fusion proteins of RUNX1 protein part with another protein, such as ETO, EVI1, and ETV6, are common in AML, CML and ALL, prevalently influencing loss of function [28,29,30].

RUNX1 expression changes are also found in solid tumours, like glioblastoma, ovarian, colon, breast, and hepatocellular carcinoma, where in tumour cells and available patient samples, it predominantly acts as a tumour suppressive. However, its oncogenic function is also documented [31,32,33,34,35,36].

*RUNX2* is involved in the progression of various human tumours by regulating cell proliferation, angiogenesis, cancer stemness, and metastasis [37]. Its expression could be deregulated by several mechanisms. Recent research has uncovered numerous somatic mutations in the *RUNX2* gene in various cancers, including missense, nonsense, and nonstop mutations and frameshift insertions and deletions [37]. In addition, amplification of the *RUNX2* gene has been observed in osteosarcoma [38] and melanoma [39]. In various tumours, RUNX2 expression is deregulated by different miRNAs, circRNAs, and regulatory proteins (reviewed in [37]).

*RUNX3* is involved in carcinogenesis by interacting with several oncogenic signalling pathways, acting as a tumour suppressor in some tumours [40,41,42,43,44] and as an oncogene in others [45,46,47]. *RUNX3* is frequently inactivated in human cancers by promoter DNA hypermethylation [40,41,42,43,44,48,49,50,51,52,53], histone modification [54,55], hemizygous deletion [50,51], and protein mislocalisation [6,43]. Although rare, inactivating somatic mutations of *RUNX3* have been detected in several cases [48,49,56]. We summarised the mechanisms underlying the deregulated expression of *RUNX* genes in Table 1.

### 1.4. The Mechanisms of Action of RUNX Proteins

Also, the co-expression of *RUNX* genes with epigenetic regulators could affect the onset of some cancer types. Researchers should pay attention to epigenetic mechanisms of *RUNX* genes’ regulation, including DNA methylation and miRNAs [22]. As epigenetic regulators, RUNX proteins cooperate with diverse coregulators and involve many signal transduction processes. In addition, they participate in chromatin landscape remodelling [10]. There is evidence that RUNX proteins can function as pioneer transcription factors that recruit various chromatin remodelling enzymes and other transcription factors to open the condensed chromatin structure and thus activate the transcription of target genes [10,22,65]. This epigenetic role of *RUNX* genes appears to play a pivotal role in both physiological and pathological conditions, including cancer [10,22]. Previous studies have shown that RUNX1 initiates chromatin remodelling in the vicinity of specific regulatory genes required for normal haematopoiesis during development [66,67], whilst its interaction with H3K4 methyltransferase and acetyltransferase E1A binding protein P300 (EP300) is implicated in leukaemia [68,69]. In physiological conditions, the interaction between RUNX2 and histone deacetylases 6 (HDAC6) is important in osteoblast differentiation [70]. On the other hand, RUNX2 has been shown to play a role in promoting the epithelial–mesenchymal transition (EMT) process in a colon cancer cell line by modulating the chromatin landscape and activating EMT-associated genes [71]. Similarly, RUNX3 is involved in cell cycle progression by recruiting chromatin-remodelling factors and cell cycle regulators, activating cell cycle restriction (R) point-associated genes [72].

The previously mentioned *RUNX* genes functions indicate that they are of great importance for further investigation of HCC. With this in mind, this review collects the existing data and questions the possibility of including *RUNX* genes as the diagnostic, predictive, or prognostic biomarkers in HCC treatment.

## 2. *RUNX1* in HCC

The *RUNX1* gene belongs to a Runt-related gene family on chromosome 21, coding for the RUNX1 protein [73]. The researchers characterised the *RUNX1* gene for the first time in chromosomal translocation t (8;21) of the acute myeloid leukaemia gene 1 (*AML1)* in AML cancer patients [74]. Two promoters, P1 (distal) and P2 (proximal), regulate the transcription of the *RUNX1* gene, forming two isoforms that differ in the first exon [75]. The researchers have discovered twelve transcription variants of the RUNX1 mRNA [7].

### 2.1. RUNX1 Role in HCC

The role of *RUNX1* in solid tumours is controversial, acting in two opposite ways. It can impede or promote carcinogenesis, as reviewed in [7,76]. Databases that collect transcriptomic studies and link data on the genomic and clinical parameters with various cancer groups show controversial results on the RUNX1 expression. According to the Gent2 and TIMER2.0 databases, there was a significantly higher expression of RUNX1 transcript in hepatocellular carcinoma compared to normal tissue [77,78]. However, the UCSC database shows the opposite results [79]. It has an upgraded version [80]. The RUNX1 expression was decreased in hepatocellular carcinoma. That was consistent with the work of Miyagawa and colleagues, who noticed that RUNX1 mRNA was 76% and 47% lower in HCC and cirrhotic tissue than in normal tissue. Also, there was a significant decrease in RUNX1 mRNA in HCC compared to cirrhotic liver samples [81]. Liu and colleagues also noticed the RUNX1 transcript and protein expression decreased in HCC patients’ samples and cell cultures compared to paratumor controls [32]. By transfecting RUNX1-expressing vectors into liver cancer cells, Liu and colleagues found that RUNX1 negatively influenced tumour cell potential of metastasis and proliferation. They assumed that RUNX1 influenced EMT and its factors. In RUNX1 overexpressed cells, they noticed Vimentin and MMP2 expression decreased, and E cadherin increased, indicating an inhibitory role of RUNX1 in the EMT process [32]. On the other hand, RUNX1 influences the upregulation of collagen type IV alpha 1 chain (COL4A1). The COL4A1 stimulated the growth and expansion of HCC cells, involving the Fak-Src signalling pathway [82].

**Figure 2:**
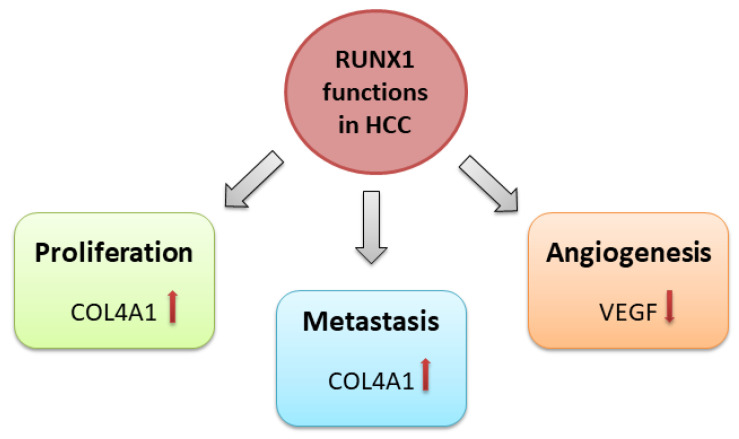
Roles of *RUNX1* in hepatocellular carcinoma. COL4A1↑-Collagen type IV alpha 1 chain increases; VEGF↓-Vascular endothelial growth factor decreases.

One of the crucial processes in cancer progression is angiogenesis. In hematopoietic cell differentiation from hemogenic endothelium cells, *RUNX1* has a vital role. The RUNX1-deficient mice lack hematopoiesis and angiogenesis [83]. Added exogenously, IGFBP-3 inhibited RUNX1-promoted angiogenesis dose-dependently [84]. Thus, we can conclude that *RUNX1* has a cardinal role in angiogenic differentiation and vascularisation. Vascular endothelial growth factor (VEGF) is an angiogenesis modulator in a cancer cell environment and the negative prognostic factor for acute myeloid leukaemia. In the HCC cell culture, Elst and colleagues found that RUNX1 inhibited VEGF expression [85]. Liu and colleagues confirmed these results on HCC patients’ samples and cell lines. They observed *VEGF* expression decreased and RUNX1 expression increased in HCC patients’ samples. The HCC cell lines with increased RUNX1 expression exhibited VEGF expression decline, too [32]. They concluded that both molecules, VEGF and RUNX1, could be the candidates for the molecularly targeted HCC treatment. Figure 2 shows the impact of *RUNX1* on proliferation, metastasis, and angiogenesis in HCC.

### 2.2. RUNX1 and miRNAs

There are not many studies of epigenetic processes involving the *RUNX1* gene. Tuo and colleagues noticed the hypomethylation of the *RUNX1* promoter in hepatocellular carcinoma [86]. Some studies show the association between several miRNAs and RUNX1 in HCC (Table 2). Transcript 1 of RUNX1 (RUNX1-IT1) is a long non-coding sequence of an RNA transcript of the *RUNX1* gene [87]. Yan and colleagues noticed the RUNX1 expression decrease in the HCC patients’ samples, and the knocking-down of RUNX1-IT1 increased the proliferation and reduced apoptosis in HCC cells [88]. Sun and colleagues observed the association of decreased RUNX1-IT1 expression with shorter DFS and OS. RUNX1-IT1 binds mir-632, competing with the other RNAs in HCC cells for target gene GSK-3β binding and modulating the WNT/β-catenin signalling cascade. Added hypoxia-prompted histone deacetylase 3 (HDAC3) in HCC cells reduced the RUNX1-IT1 expression. They concluded that the goal of HCC therapy should be to activate RUNX1-IT1 [89]. On the other hand, Vivacqua and colleagues noticed that oestrogen receptor agonists, such as the G protein-coupled oestrogen receptor agonist (G-1) and 17β-oestradiol (E2), increased miR-144 expression in HepG2 hepatocarcinoma cells, via the G protein-coupled oestrogen receptor 1 (GPER) and the PI3K/ERK1/2/Elk1 pathway. miR-144 then downregulates RUNX1, promoting the cell cycle [90] (Table 2).

Li and colleagues found that the molecule Pam3CSK4, an agonist of Toll-like receptor 2 (TLR2), injected into mice inhibited tumour growth and reduced myeloid-derived suppressor cells (MDSCs), thereby attenuating HCC progression [99]. MDSCs participate in the formation of an immune microenvironment of the tumour. Pam3CSK4 targets RUNX1 and promotes MDSC polarisation. On the contrary, inhibiting RUNX1 resulted in tumour enlargement and shortened overall survival. Their results indicated the role of RUNX1 and TLR2 in the MDSCs’ formation, function, and polarity. Considering all this, RUNX1 and TLR2 targeting could lead to a potential mechanism of HCC immunotherapy [99].

In conclusion, RUNX1 binds target genes (VEGF and COL4A1) and involves signalling pathways of cancer proliferation, metastasis, and angiogenesis. RUNX1 also interacts with several miRNAs, for example, mir-632 and mir-144. Given the importance of RUNX1 in hepatocellular carcinoma, its potential suitability as a treatment target requires additional studies. Researchers should pay particular attention to the binding of RUNX1 to the other genes and miRNAs and its involvement in signalling pathways.

## 3. *RUNX2* in HCC

The second member of Runt-related family genes, *RUNX2*, is located on the 6p21 chromosomal region, with the function of a transcriptional regulator in human osteoblast differentiation and chondrocyte maturation [100,101]. Further experimental data indicate that *RUNX2* also has a carcinogenic function in various human malignancies [102]. As a regulatory molecule, RUNX2 has been a part of molecular networks that promote the invasive behaviour of tumours [102].

### 3.1. General Role of RUNX2 in HCC

According to literature data, the expression of RUNX2 on mRNA and/or protein level is elevated in HCC cell lines, as well as in liver tumour tissue [95,103,104], suggesting that this transcription factor has a role in hepatocarcinogenesis. Previous findings confirmed higher RUNX2 expression in HCC patients than expression detected in non-tumour tissues or healthy controls [95,103,105]. Wang and colleagues noticed that increased expression of RUNX2 significantly correlates with unfavourable clinicopathological features in HCC. These adverse features included the onset of multiple tumour nodes, higher histological grades and TNM stages, and venous invasion presence [103]. Moreover, aberrant RUNX2 expression could be an independent prognostic factor since hepatocellular carcinoma patients with high RUNX2 expression demonstrated shorter 5-year disease-free and overall survival [103,104]. Additionally, RUNX2 contributed to the HCC development regardless of the presence of HBV or HCV infections. In addition, the measured level of RUNX2 expression was not significantly impaired by the HCV or HBV existence [95].

### 3.2. RUNX2 Tumour Invasion Activity in HCC

**Figure 3:**
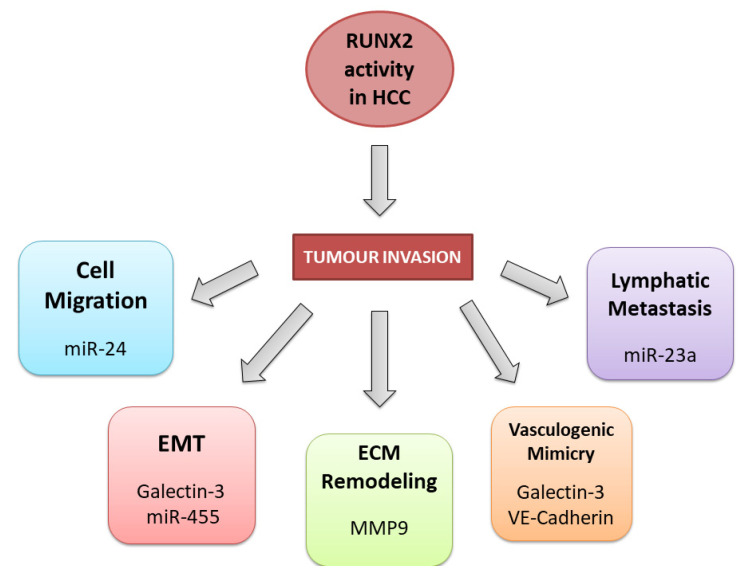
*RUNX2* oncogenic mechanisms in hepatocellular carcinoma.

The general mechanisms underlying the role of *RUNX2* in various tumour types provide directions for detailed studies on the impact of *RUNX2* on the pathogenesis of HCC. Many reports showed that increased RUNX2 expression enhances tumour cell migration and invasive properties [106,107,108,109,110,111,112]. Previous studies revealed the crucial role of the RUNX2 in the regulation of the epithelial-to-mesenchymal transition (EMT) process in many tumours [104,113], which is the first step toward tumour invasion and metastatic potential (Figure 3).

Cao and colleagues found that RUNX2 overexpression can promote EMT in HCC [104]. Elevated RUNX2 expression can also trigger vasculogenic mimicry (VM), providing a direct metastatic route to distant sites [114,115]. Experiments on HCC cell lines revealed that RUNX2 is associated with EMT and VM processes by regulating the expression of adhesion molecules such as VE-cadherin and galectin-3, which indirectly contribute to tumour cell migration and enhanced metastatic potential [104,116,117]. Moreover, RUNX2 is implicated in tissue microenvironment regulation and extracellular matrix reshaping. A previous report demonstrated that RUNX2 acted as an initiator of migration and invasion of the HCC cells in vitro by enhancing the expression of the matrix metalloproteinase 9 (MMP9) [103]. 

The level of RUNX2 expression significantly correlates with the expression level of MMP9 in hepatocellular carcinoma [103]. This association between RUNX2 and MMP9 expression levels was also detected in breast cancer [109]. Moreover, the results of two experimental studies have clarified *RUNX2’s* indirect oncogenic role in hepatocarcinogenesis. Using a gain/loss-of-function study approach, Yang and colleagues demonstrated that zinc finger protein 521 (ZNF521) strongly repressed the transcriptional activity of RUNX2 and affected RUNX2-related PI3K/AKT signalling pathways, significantly inhibiting HCC growth [118]. Moreover, ZNF521-mediated downregulation of RUNX2 also suppresses tumorigenic processes in HCC cells [118]. Moreover, the downregulated *RUNX2* gene notably decreased the HCC cells’ propagation, migration, and chemoresistance [105]. This study specifically examined the role of RUNX2 in the NUPR1/RELB/IER3 signalling cascade as a suggested molecular mechanism underlying HCC development and response to sorafenib treatment [105].

### 3.3. RUNX2 and Non-Coding RNAs in HCC

Previous studies showed that multiple microRNAs could be differentially expressed in HCC, directly or indirectly affecting RUNX2 expression and activity (Table 2). *RUNX2* might be directly repressed by the miR-455 molecule in human HCC samples [91], which has already demonstrated tumour-suppressive properties [119,120]. Further gain/loss-of-function analyses showed that in HCC cells, miR-455 regulates the process of RUNX2 accumulation in vitro, which significantly suppresses cell migration abilities [91]. Additionally, several miRNAs can regulate RUNX2 expression by directly binding to the *RUNX2* gene 3′-UTR region [121,122]. Wang and colleagues suggested that miR-196a could have a significant role in HCC development through the *RUNX2* upregulation, which in HCC cell lines produced higher osteopontin levels as a consequence [92]. Osteopontin is a well-known bone marrow-produced protein that regulates bone regeneration, although previous reports indicate that this protein also contributes to cancer metastasis [123]. In addition, *RUNX2* may be entangled in the HCC development by directing the expression level regulation of several miRNAs. Wang and colleagues investigated the mechanism underlying the increased level of O-GlcNAc transferase, which enhances tumour cell migratory abilities and HCC invasive capacities [93]. Their results showed that RUNX2 indirectly affects OGT expression via transcriptional activation of miR-24 by binding to its promoter [93]. In another study on mouse hepatoma cells, RUNX2 binds to the *miR-23a* gene’s promoter and indirectly promotes lymphatic metastasis by targeting the Mgat3 glycosyltransferase directly affecting the glycosylation process on the cell surface [94].

Increasing evidence suggests that RUNX2 can interact with long non-coding RNAs (lncRNAs), contributing to hepatocellular carcinogenesis (Table 1). For example, the lncRNA called HAND2-AS plays a tumour-suppressive role in liver cancer and prohibits hepatoma cancer cell proliferation by decreasing the expression level of RUNX2 [95]. In another study, RUNX2 and transcriptional regulator YAP inhibit the expression level of lncRNA annotated MT1DP, demonstrating tumour-suppressive behaviour in hepatocellular carcinoma [96]. However, detailed analyses are necessary to clarify the correlation between RUNX2 and different lncRNAs and their synergistic effect on liver carcinogenesis.

RUNX2, a unique transcription factor, exhibits a crucial oncogenic role in hepatocellular carcinoma. Moreover, we should consider RUNX2 aberrant expression as a novel prognostic indicator in HCC. Studies on RUNX2-related regulatory mechanisms hint at its pro-invasive functions in HCC by reshaping the tumour microenvironment, making RUNX2 a potential therapeutic target for blocking metastasis and further disease progression. Since the RUNX2 transcription regulator is implicated in many signalling pathways and interacts with multiple regulatory molecules like microRNAs and lncRNAs, more in-depth studies to clarify its role in the molecular pathology of hepatocellular carcinoma are needed.

## 4. *RUNX3* in HCC

The third member of the Runt-related gene family, *RUNX3,* is located in the chromosomal region 1p36–35 [49]. *RUNX3* has initially been reported as a tumour suppressor in gastric cancer [41]. Subsequent studies confirmed its tumour-suppressive role in some of the most common cancer types in humans, including colorectal [42], prostate [43], breast [44], lung cancer [40], and melanoma [46]. On the other hand, *RUNX3* has been shown to act as an oncogene and promote tumour development in ovarian [47], head and neck [45], and pancreatic carcinoma [124]. This dualistic function of *RUNX3* is cell-context dependent [125]. 

*RUNX3* is required for normal liver development, while its loss is associated with hepatocellular carcinogenesis, where it acts as a tumour suppressor [18,50]. *RUNX3* gene expression is decreased in up to 80% of HCCs, predominantly due to promoter methylation. The loss of heterozygosity (LOH) was also observed in several cases [51,64]. In two meta-studies, *RUNX3* hypermethylation has been shown to occur early in hepatocarcinogenesis, including premalignant conditions like liver fibrosis and cirrhosis, with the highest frequencies being reported in HCC [126,127]. 

### 4.1. General Role of RUNX3 in HCC

**Figure 4:**
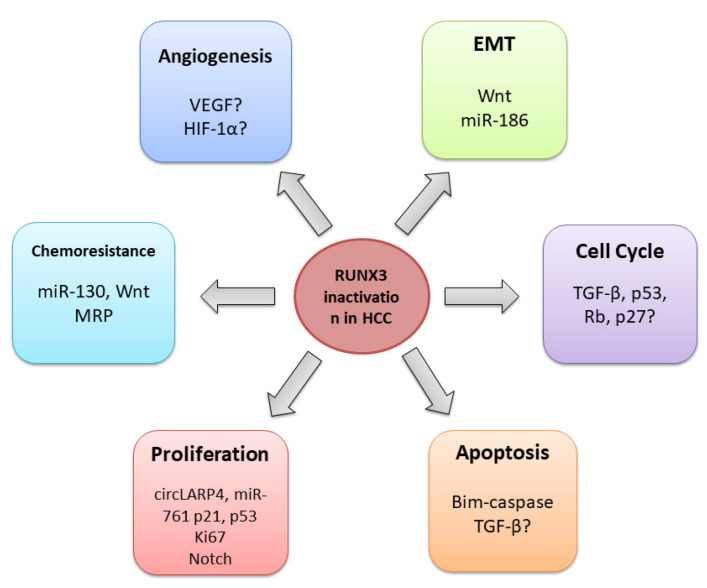
The downregulation of *RUNX3* in HCC.

Several studies have shown that *RUNX3* inactivation is cardinal for the initiation and progression of HCC [98,126,127,128,129,130]. As a multifunctional transcription factor, RUNX3 is implicated in diverse signalling pathways and cellular processes, thereby exerting multiple effects on tumour suppression [131,132]. According to current knowledge, RUNX3 participates in the regulation of the cell cycle [133], proliferation and apoptosis [134], angiogenesis [18], and EMT [98,135]. Its loss is also related to chemoresistance [136,137] (Figure 4).

### 4.2. RUNX3 Regulates Cell Cycle, Proliferation, and Apoptosis 

Dysregulation of the cell cycle is a prime event in hepatocarcinogenesis. RUNX3 may play a pivotal role in this process by employing diverse mechanisms [134]. Earlier studies on gastric epithelial cells demonstrated that RUNX3 regulates the cell cycle by interacting with p21, p27, and cyclin D1 proteins [138,139,140]. Further research revealed that RUNX3 induces the expression of the *ARF* and *CDKN1A* cell cycle regulators by interaction with BRD2 and pRB proteins [40,133]. Moreover, it has recently been shown that RUNX3 activates the cell cycle restriction (R) point-associated genes by recruitment of chromatin-remodelling complex, histone modifiers, and cell-cycle regulators to form the RUNX3-containing activator complex, which opens chromatin structure in the vicinity of target genes [72]. Whether RUNX3 exerts such a function in HCC remains to be elucidated.

As the major component of the transforming growth factor-beta signalling (TGF-β) pathway [8,141], RUNX3 can stop cell proliferation, inducing a p21 cell-cycle inhibitor [140]. Similarly, it can suppress apoptosis by inducing apoptosis initiator Bim, as has been shown on gastric cancer cell lines [142].

Another study on human HCC cell lines demonstrated that RUNX3 could induce apoptosis through the Bim–caspase pathway, even in the absence of TGF-β [50]. RUNX3 also regulates the TGF-β-mediated growth arrest by the induction of CDK inhibitors and/or the repression of the c-*Myc* proto-oncogene [143,144].

Another study in mice showed that proliferation marker Ki67 was more frequently observed in the *RUNX3* knockout liver cells than in wild-type cells, which further confirmed the role of RUNX3 in hepatocyte proliferation regulation [18]. RUNX3 has been reported to control cellular senescence, a potent anti-cancer mechanism that prevents the proliferation of potentially cancerous cells [145]. A recent study on human HCC samples and cell lines demonstrated that RUNX3 could modulate the expression of key markers of cellular senescence, p53 and p21, via the circLARP4/miR-761/RUNX3 signalling axis [97] (Table 2). As a competing endogenous RNA, the circLARP4 harbours miR-761, abrogating its inhibitory effect on the *RUNX3* gene. RUNX3 subsequently activates the p53/p21 signalling pathway and enhances the downstream senescence phenotype in HCC [97]. 

In addition, evidence suggests that RUNX3 can regulate cell cycle and apoptosis through the Wnt/β-catenin signalling pathway, whose oncogenic activation is a usual event in HCC [52,146,147]. RUNX3 directly interacts with the Wnt transcription factor, the TCF4-β-catenin complex, and thus inhibits the expression of Wnt target genes, *c*-*Myc* and *cyclin D*, regulators of apoptosis and the cell cycle, respectively [53,63,131]. 

Oncogenic activation of the Notch signalling pathway is also implicated in hepatocyte growth and proliferation [148,149]. Gao and colleagues have shown that RUNX3 can suppress oncogenic Notch signalling through direct interaction with the intracellular domain of the Notch1 protein in HCC cell lines [132]. Further studies revealed that RUNX3 decreases jagged-1 (JAG1) mRNA and thus inhibits JAG1-mediated Notch signalling in HCC [148,150]. Moreover, RUNX3 has been reported to inhibit the transcription of HES1, the Notch target gene implicated in stemness, metastasis, and chemoresistance regulation in cancer [132]. Given that, affecting Notch1 signalling by *RUNX3* reactivation might be a promising therapeutic approach for the HCC treatment. 

### 4.3. RUNX3 in the Angiogenesis Regulation 

A crucial tumour-suppressive role of RUNX3 is angiogenesis prevention and tumour invasion. A recent study revealed that after the HCC therapeutic drug’s application, sorafenib, RUNX3 suppressed VEGF expression in HCC, which was associated with reduced tumour growth [151]. A previous study on gastric cancer cells demonstrated that RUNX3 destabilised hypoxia-inducible factor HIF-1*α* in the hypoxic microenvironment, thus inhibiting angiogenesis [152]. Additional research is necessary to confirm whether this mechanism exists in HCC, as inhibition of angiogenesis is an important therapeutic strategy for the prevention of HCC progression [153].

### 4.4. RUNX3 and Epithelial-Mesenchymal Transition

Previous studies have shown that the loss of RUNX3 contributes to EMT, a crucial process related to metastasis, chemoresistance, and tumour stemness [154,155]. In vitro experiments demonstrated that RUNX3 repressed tumour metastasis and invasion by upregulating E-cadherin through the miR-186/E-cadherin/EMT axis [98,156] (Table 2). In addition, experiments on human HCC cell lines revealed that the loss of RUNX3 supports pro-oncogenic TGF-β signalling through the upregulation of EMT genes and that RUNX3 can also suppress EMT via the inhibition of Wnt signalling [135]. Given its crucial role in carcinogenesis, targeting EMT by re-expressing *RUNX3* could be another potential therapeutic approach for treating HCC patients.

### 4.5. RUNX3 and Chemoresistance 

A study on human HCC samples and cell lines demonstrated that *RUNX3* could be downregulated by overexpression of miR-130 through the miR-130a/RUNX3/Wnt signalling pathway. This mechanism was associated with increased chemoresistance to cisplatin [137]. Studies in gastric [157] and cervical cancer [158] demonstrated that miR-130 directly binds to the *RUNX3* and thus inhibits its expression. Accordingly, restoration of *RUNX3* expression by targeting miR-130 could be a potential approach to overcome chemotherapy resistance in HCC patients. 

Researchers also demonstrated that the loss of RUNX3 contributes to 5-fluorouracil (5-FU) and cisplatin (CDDP) resistance in HCC cell lines and patients through increased expression of multidrug resistance-associated proteins (MRP) [136]. Drug resistance is an extensive obstacle to the successful treatment of HCC. Therefore, additional research is necessary to address this issue and develop more efficient treatment approaches. 

All concerning, RUNX3 appears to be involved in hepatocarcinogenesis at distinct stages, from initiation to progression and metastasis. Thus, its potential clinical application might have a wide range. However, given that many results are on cell lines, further studies on HCC patients are needed for a complete understanding of the significance of RUNX3 in HCC.

## 5. Conclusions and Future Directions

Despite considerable advances in cancer diagnosis and treatment, HCC remains one of the most common and hard-to-treat human cancers. Revealing the essential molecular processes underlying hepatocarcinogenesis is crucial for establishing reliable diagnostic, prognostic, and therapeutic markers. *RUNX* genes are often deregulated in HCC, exerting complex and conflicting functions. The role of *RUNX1* is still contradictory, as there are reports of its tumour-suppressive but also oncogenic role in HCC. According to current knowledge, *RUNX2* acts as an oncogene and is related to the more aggressive forms of the disease, whereas *RUNX3* exerts a tumour-suppressive role and could be used as a biomarker for early HCC detection. All three genes could serve as therapeutic targets. However, a deeper understanding of the relationship between different RUNX family members and the signalling pathways they are involved in, considering the cell-specific microenvironment, is necessary for effective HCC therapeutic strategy development.

As previously mentioned, treatment of HCC remained clinically demanding due to its highly drug-resistant nature. The first-line therapeutics barely prolong overall survival, although recent studies provide evidence that sorafenib, in combination with other active components, may achieve a more effective HCC response [159]. Synthetic lethality (SL), the concept where concurrent losses of two genes are lethal to a cell, while a single gene loss does not affect cell viability, emerged as the promising HCC treatment strategy in recent years [159,160]. By high throughput genome analyses, several HCC driver mutations have been revealed recently [161,162,163], including *p53* mutation as the most common genetic change detected in 30% of HCC cases [164]. Although therapeutic targeting of the *p53* tumour suppressor may be challenging, searching for a suitable synthetic lethality *p53* gene partner could be a promising approach in HCC individualised treatment development [165]. Considering the role of RUNX-p53 interaction in carcinogenesis in general, Bae et al. proposed a two-step tumour-suppressive model in which RUNX proteins prevent adenoma formation at first, whilst p53 functions at later stages to prevent adenocarcinoma [166]. In the regulation of the DNA damage response, both RUNX1 and RUNX3 form a complex with p53 and promote the transactivation of p53 target genes (*BAX*, *PUMA*, *NOXA*, and *p21*), whilst the interaction of RUNX2 with p53 suppresses the transactivation of p53 target genes such as *p21*, *WAF1*, and *BAX* [167], so the potential SL interaction between *p53* and *RUNX* genes in HCC requires further investigation. A recent comprehensive bioinformatics study tested 14 tumour-suppressor and 3194 druggable genes (including *RUNX1*, *RUNX2*, and *RUNX3*) using functional similarity and differential gene expression analysis for SL interaction identification in HCC, and a total of 272 potential SL pairs were revealed, whilst *RUNX* genes did not pass initial screening tests [165]. However, more detailed computational and experimental analyses of potential RUNX synthetic lethality networks, simultaneously with RUNX-based target treatment development, have to be future directions toward individualised therapy of HCC.

Increasing evidence suggests that *RUNX* genes act as epigenetic modulators that interact with other chromatin landscape regulators to activate or repress the transcription of target genes [136]. Since normal epigenetic patterns are altered in all types of human cancers, it would be of great interest to investigate interactions between RUNX proteins and other epigenetic regulators, especially in HCC. This could potentially provide an avenue for epigenetic therapy.

As previously stated, there are several possible ways of potential *RUNX1* use in future therapy of HCC. A possible way is targeting long coding intronic transcript 1 of *RUNX1*, a hypoxia regulator in HCC, which modulates the WNT/β-catenin signalling cascade [89]. The other is direct *RUNX1* targeting, as its involvement is documented in myeloid-derived suppressor cell formation [99], GPER, and the PI3K/ERK1/2/Elk1 pathway signalling cascade [90]. The combination of *RUNX1* and one of its targets, VEGF, which is found to be downregulated by RUNX1, could also be used in future targeted treatment [85]. Most of the findings about *RUNX1* are on HCC cells, and extensive work is needed on the clinical level to examine the treatment potential of *RUNX1*. Also, the mechanisms of RUNX1 function, which determine its role in the specific cellular context, depend on the signalling cascades activated at the given moment. 

Considering *RUNX2* and its overexpression and oncogenic function in HCC, the design of a highly selective chemical or RNA-based inhibitor is a desirable approach. According to the data available on the PHAROS web interface for exploring target/ligand interactions [168], for the query ”RUNX2”, currently, there are no approved drugs or active ligands (ChEMBL compounds with an activity cutoff of <30 nM) available, so clinical trials focusing on testing RUNX2 based-drugs in HCC and in tumours in general are still an unexplored field.

In contrast to *RUNX1* and *RUNX2*, *RUNX3* is inactivated in most HCC cases almost exclusively by promoter methylation. Therefore, its function could potentially be restored by demethylation agents (e.g., azacytidine and decitabine) and HAD inhibitors. The effects of the re-expressed *RUNX3* gene on tumour progression remain to be elucidated. Moreover, there is evidence that *RUNX3* methylation is higher in HCV-related HCC than in non-HCV-related HCC [169]. HCV is known to be involved in hepatocarcinogenesis through a complex epigenetic network, including altered host DNA methylation patterns and deregulated expression of histone modifiers and specific miRNAs (reviewed in [170]). Future studies should also focus on the molecular characterisation of HCC in the contest of their specific aetiology. Taken together, a comprehensive analysis of the genetic and epigenetic molecular mechanisms underlying *RUNX* gene deregulation in HCC could improve current therapy approaches.

## Figures and Tables

**Table 1:** The mechanisms underlying the deregulated expression of *RUNX* genes.

Gene	Regulation Mechanisms	Disease	References
*RUNX1*	Point mutations	AML, FPD/AML	[57]
	Frameshift mutations	AML FPD	[27,58] [57]
	Translocations	AML CML ALL	[29] [30] [26]
	Decreased expression of RUNX1 and increased of VEGF	HCC	[32]
	Increased expression	Colorectal cancer Glioblastoma Epithelial ovarian cancer	[33] [34] [35] [31]
	Loss of RUNX1	Breast cancer cell lines	[36]
*RUNX2*	Missense, nonsense, nonstop, deletions, and frameshift	Different types of cancers	[37]
	Gene amplification	Osteosarcoma Melanoma	[38] [39]
	Increased expression	Clear cell renal cell carcinoma Colorectal cancer Bladder cancer Lung adenocarcinoma	[59] [60] [61] [62]
*RUNX3*	Promoter hypermethylation	Gastric cancer Sporadic colon cancer Prostate cancer Breast cancer Lung adenocarcinoma Melanoma Bladder cancer HCC	[41,63] [42,53] [43] [44] [40] [46] [48] [51,64]
	Histone modification	Gastric cancer cells	[55]
	LOH	HCC	[51]
	Protein mislocalisation	Breast cancer	[44]
	R122C point mutation	Gastric cancer	[41]
	L89P, P102T, A119D, and M128V	Bladder cancer	[48]

AML—acute myeloid leukaemia; FPD/AML—familial platelet disorder with acute myeloid leukaemia; CML—chronic myeloid leukaemia; ALL—acute lymphoblastic leukaemia; and HCC—hepatocellular carcinoma.

**Table 2:** The link between miRNAs or lncRNAs and *RUNX* genes in HCC.

Gene	miRNA(s) or lncRNA(s)	References
*RUNX1*	miR-632	[89]
	miR-144	[90]
*RUNX2*	miR-455	[91]
	miR-196	[92]
	miR-24	[93]
	miR-23a	[94]
	lncRNA HAND2 antisense RNA 1 (HAND2-AS1)	[95]
	lncRNA metallothionein 1D, pseudogene (MT1DP)	[96]
*RUNX3*	miR-761	[97]
	miR-130	[98]

## Data Availability

No new data were created or analysed in this study. Data sharing does not apply to this article.

## Abbreviations

HCC	Hepatocellular carcinoma;
RUNX	Runt-related transcription factors;
HBV	Hepatitis B Virus;
HCV	Hepatitis C Virus;
RHD	Runt homology domain;
miRNA	Micro ribonucleic acids;
lncRNAs	Long non-coding ribonucleic acids

## References

[1] Asafo-Agyei K.O., Samant H. (2023). Hepatocellular Carcinoma. StatPearls.

[2] Llovet J.M., Kelley R.K., Villanueva A., Singal A.G., Pikarsky E., Roayaie S., Lencioni R., Koike K., Zucman-Rossi J., Finn R.S. (2021). Hepatocellular Carcinoma. Nat. Rev. Dis. Prim..

[3] Balogh J., Victor D., Asham E.H., Burroughs S.G., Boktour M., Saharia A., Li X., Ghobrial M., Monsour H. (2016). Hepatocellular Carcinoma: A Review. J. Hepatocell. Carcinoma.

[4] Sim H.-W., Knox J. (2018). Hepatocellular Carcinoma in the Era of Immunotherapy. Curr. Probl. Cancer.

[5] Suresh D., Srinivas A.N., Prashant A., Harikumar K.B., Kumar D.P. (2023). Therapeutic Options in Hepatocellular Carcinoma: A Comprehensive Review. Clin. Exp. Med..

[6] Chuang L.S.H., Ito K., Ito Y. (2013). RUNX Family: Regulation and Diversification of Roles through Interacting Proteins. Int. J. Cancer.

[7] Otálora-Otálora B.A., Henríquez B., López-Kleine L., Rojas A. (2019). RUNX Family: Oncogenes or Tumor Suppressors (Review). Oncol. Rep..

[8] Ito Y., Bae S.-C., Chuang L.S.H. (2015). The RUNX Family: Developmental Regulators in Cancer. Nat. Rev. Cancer.

[9] Levanon D., Groner Y. (2004). Structure and Regulated Expression of Mammalian RUNX Genes. Oncogene.

[10] Yi H., He Y., Zhu Q., Fang L. (2022). RUNX Proteins as Epigenetic Modulators in Cancer. Cells.

[11] Mevel R., Draper J.E., Lie-a-Ling M., Kouskoff V., Lacaud G. (2019). RUNX Transcription Factors: Orchestrators of Development. Development.

[12] Darnell J.E. (2002). Transcription Factors as Targets for Cancer Therapy. Nat. Rev. Cancer.

[13] Sood R., Kamikubo Y., Liu P. (2017). Role of RUNX1 in Hematological Malignancies. Blood.

[14] Ito Y. (2004). Oncogenic Potential of the RUNX Gene Family: ‘Overview’. Oncogene.

[15] Lee Y.M. (2022). RUNX Family in Hypoxic Microenvironment and Angiogenesis in Cancers. Cells.

[16] Yamagata T., Maki K., Mitani K. (2005). Runx1/AML1 in Normal and Abnormal Hematopoiesis. Int. J. Hematol..

[17] Dalle Carbonare L., Innamorati G., Valenti M.T. (2012). Transcription Factor Runx2 and Its Application to Bone Tissue Engineering. Stem Cell Rev. Rep..

[18] Lee J.-M., Lee D.-J., Bae S.-C., Jung H.-S. (2013). Abnormal Liver Differentiation and Excessive Angiogenesis in Mice Lacking Runx3. Histochem. Cell Biol..

[19] Wang J.W., Stifani S., Groner Y., Ito Y., Liu P., Neil J.C., Speck N.A., Van Wijnen A. (2017). Roles of Runx Genes in Nervous System Development. RUNX Proteins in Development and Cancer.

[20] Guilliams M., Bonnardel J., Haest B., Vanderborght B., Wagner C., Remmerie A., Bujko A., Martens L., Thoné T., Browaeys R. (2022). Spatial Proteogenomics Reveals Distinct and Evolutionarily Conserved Hepatic Macrophage Niches. Cell.

[21] Human All Liver Cells. https://www.livercellatlas.org/umap-humanAll.php.

[22] Pan S., Sun S., Liu B., Hou Y. (2022). Pan-Cancer Landscape of the RUNX Protein Family Reveals Their Potential as Carcinogenic Biomarkers and the Mechanisms Underlying Their Action. J. Transl. Intern. Med..

[23] Papaemmanuil E., Gerstung M., Bullinger L., Gaidzik V.I., Paschka P., Roberts N.D., Potter N.E., Heuser M., Thol F., Bolli N. (2016). Genomic Classification and Prognosis in Acute Myeloid Leukemia. N. Engl. J. Med..

[24] Kellaway S.G., Keane P., Edginton-White B., Regha K., Kennett E., Bonifer C. (2021). Different Mutant RUNX1 Oncoproteins Program Alternate Haematopoietic Differentiation Trajectories. Life Sci. Alliance.

[25] Osato M. (2004). Point Mutations in the RUNX1/AML1 Gene: Another Actor in RUNX Leukemia. Oncogene.

[26] Döhner H., Estey E., Grimwade D., Amadori S., Appelbaum F.R., Büchner T., Dombret H., Ebert B.L., Fenaux P., Larson R.A. (2017). Diagnosis and Management of AML in Adults: 2017 ELN Recommendations from an International Expert Panel. Blood.

[27] Gaidzik V.I., Teleanu V., Papaemmanuil E., Weber D., Paschka P., Hahn J., Wallrabenstein T., Kolbinger B., Köhne C.H., Horst H.A. (2016). RUNX1 Mutations in Acute Myeloid Leukemia Are Associated with Distinct Clinico-Pathologic and Genetic Features. Leukemia.

[28] Romana S., Mauchauffe M., Le Coniat M., Chumakov I., Le Paslier D., Berger R., Bernard O. (1995). The t(12;21) of Acute Lymphoblastic Leukemia Results in a Tel-AML1 Gene Fusion. Blood.

[29] Miyoshi H., Kozu T., Shimizu K., Enomoto K., Maseki N., Kaneko Y., Kamada N., Ohki M. (1993). The t(8;21) Translocation in Acute Myeloid Leukemia Results in Production of an AML1-MTG8 Fusion Transcript. EMBO J..

[30] Mitani K., Ogawa S., Tanaka T., Miyoshi H., Kurokawa M., Mano H., Yazaki Y., Ohki M., Hirai H. (1994). Generation of the AML1-EVI-1 Fusion Gene in the t(3;21)(Q26;Q22) Causes Blastic Crisis in Chronic Myelocytic Leukemia. EMBO J..

[31] Keita M., Bachvarova M., Morin C., Plante M., Gregoire J., Renaud M.-C., Sebastianelli A., Trinh X.B., Bachvarov D. (2013). The RUNX1 Transcription Factor Is Expressed in Serous Epithelial Ovarian Carcinoma and Contributes to Cell Proliferation, Migration and Invasion. Cell Cycle.

[32] Liu C., Xu D., Xue B., Liu B., Li J., Huang J. (2020). Upregulation of RUNX1 Suppresses Proliferation and Migration through Repressing VEGFA Expression in Hepatocellular Carcinoma. Pathol. Oncol. Res..

[33] Li Q., Lai Q., He C., Fang Y., Yan Q., Zhang Y., Wang X., Gu C., Wang Y., Ye L. (2019). RUNX1 Promotes Tumour Metastasis by Activating the Wnt/β-Catenin Signalling Pathway and EMT in Colorectal Cancer. J. Exp. Clin. Cancer Res..

[34] Lu C., Yang Z., Yu D., Lin J., Cai W. (2020). RUNX1 Regulates TGF-β Induced Migration and EMT in Colorectal Cancer. Pathol. Res. Pract..

[35] Sangpairoj K., Vivithanaporn P., Apisawetakan S., Chongthammakun S., Sobhon P., Chaithirayanon K. (2017). RUNX1 Regulates Migration, Invasion, and Angiogenesis via P38 MAPK Pathway in Human Glioblastoma. Cell. Mol. Neurobiol..

[36] Fritz A.J., Hong D., Boyd J., Kost J., Finstaad K.H., Fitzgerald M.P., Hanna S., Abuarqoub A.H., Malik M., Bushweller J. (2020). RUNX1 and RUNX2 Transcription Factors Function in Opposing Roles to Regulate Breast Cancer Stem Cells. J. Cell. Physiol..

[37] Lin T.-C. (2023). RUNX2 and Cancer. Int. J. Mol. Sci..

[38] Nie J.-H., Yang T., Li H., Ye H.-S., Zhong G.-Q., Li T.-T., Zhang C., Huang W.-H., Xiao J., Li Z. (2021). Identification of GPC3 Mutation and Upregulation in a Multidrug Resistant Osteosarcoma and Its Spheroids as Therapeutic Target. J. Bone Oncol..

[39] Lake S.L., Jmor F., Dopierala J., Taktak A.F.G., Coupland S.E., Damato B.E. (2011). Multiplex Ligation-Dependent Probe Amplification of Conjunctival Melanoma Reveals Common *BRAF* V600E Gene Mutation and Gene Copy Number Changes. Invest. Ophthalmol. Vis. Sci..

[40] Lee Y.-S., Lee J.-W., Jang J.-W., Chi X.-Z., Kim J.-H., Li Y.-H., Kim M.-K., Kim D.-M., Choi B.-S., Kim E.-G. (2013). Runx3 Inactivation Is a Crucial Early Event in the Development of Lung Adenocarcinoma. Cancer Cell.

[41] Li Q.-L., Ito K., Sakakura C., Fukamachi H., Inoue K., Chi X.-Z., Lee K.-Y., Nomura S., Lee C.-W., Han S.-B. (2002). Causal Relationship between the Loss of RUNX3 Expression and Gastric Cancer. Cell.

[42] Goel A., Arnold C.N., Tassone P., Chang D.K., Niedzwiecki D., Dowell J.M., Wasserman L., Compton C., Mayer R.J., Bertagnolli M.M. (2004). Epigenetic Inactivation OfRUNX3 in Microsatellite Unstable Sporadic Colon Cancers. Int. J. Cancer.

[43] Kang G.H., Lee S., Lee H.J., Hwang K.S. (2004). Aberrant CpG Island Hypermethylation of Multiple Genes in Prostate Cancer and Prostatic Intraepithelial Neoplasia: CpG Island Methylation in Prostate Cancer and PIN. J. Pathol..

[44] Lau Q.C., Raja E., Salto-Tellez M., Liu Q., Ito K., Inoue M., Putti T.C., Loh M., Ko T.K., Huang C. (2006). RUNX3 Is Frequently Inactivated by Dual Mechanisms of Protein Mislocalization and Promoter Hypermethylation in Breast Cancer. Cancer Res..

[45] Tsunematsu T., Kudo Y., Iizuka S., Ogawa I., Fujita T., Kurihara H., Abiko Y., Takata T. (2009). RUNX3 Has an Oncogenic Role in Head and Neck Cancer. PLoS ONE.

[46] Zhang Z., Chen G., Cheng Y., Martinka M., Li G. (2011). Prognostic Significance of RUNX3 Expression in Human Melanoma: RUNX3 in Melanoma Prognosis. Cancer.

[47] Nevadunsky N.S., Barbieri J.S., Kwong J., Merritt M.A., Welch W.R., Berkowitz R.S., Mok S.C. (2009). RUNX3 Protein Is Overexpressed in Human Epithelial Ovarian Cancer. Gynecol. Oncol..

[48] Kim W.-J., Kim E.-J., Jeong P., Quan C., Kim J., Li Q.-L., Yang J.-O., Ito Y., Bae S.-C. (2005). *RUNX3* Inactivation by Point Mutations and Aberrant DNA Methylation in Bladder Tumors. Cancer Res..

[49] Lund A.H., Van Lohuizen M. (2002). RUNX: A Trilogy of Cancer Genes. Cancer Cell.

[50] Nakanishi Y., Shiraha H., Nishina S., Tanaka S., Matsubara M., Horiguchi S., Iwamuro M., Takaoka N., Uemura M., Kuwaki K. (2011). Loss of Runt-Related Transcription Factor 3 Expression Leads Hepatocellular Carcinoma Cells to Escape Apoptosis. BMC Cancer.

[51] Mori T., Nomoto S., Koshikawa K., Fujii T., Sakai M., Nishikawa Y., Inoue S., Takeda S., Kaneko T., Nakao A. (2005). Decreased Expression and Frequent Allelic Inactivation of the RUNX3 Gene at 1p36 in Human Hepatocellular Carcinoma. Liver Int..

[52] Steinhart Z., Angers S. (2018). Wnt Signaling in Development and Tissue Homeostasis. Development.

[53] Ito K., Lim A.C.-B., Salto-Tellez M., Motoda L., Osato M., Chuang L.S.H., Lee C.W.L., Voon D.C.-C., Koo J.K.W., Wang H. (2008). RUNX3 Attenuates β-Catenin/T Cell Factors in Intestinal Tumorigenesis. Cancer Cell.

[54] Lee Y.M. (2011). Control of RUNX3 by Histone Methyltransferases. J. Cell. Biochem..

[55] Lee S.H., Kim J., Kim W.-H., Lee Y.M. (2009). Hypoxic Silencing of Tumor Suppressor RUNX3 by Histone Modification in Gastric Cancer Cells. Oncogene.

[56] cBioPortal For Cancer Genomics. https://www.cbioportal.org/.

[57] Song W.-J., Sullivan M.G., Legare R.D., Hutchings S., Tan X., Kufrin D., Ratajczak J., Resende I.C., Haworth C., Hock R. (1999). Haploinsufficiency of CBFA2 Causes Familial Thrombocytopenia with Propensity to Develop Acute Myelogenous Leukaemia. Nat. Genet..

[58] Mendler J.H., Maharry K., Radmacher M.D., Mrózek K., Becker H., Metzeler K.H., Schwind S., Whitman S.P., Khalife J., Kohlschmidt J. (2012). RUNX1 Mutations Are Associated with Poor Outcome in Younger and Older Patients with Cytogenetically Normal Acute Myeloid Leukemia and with Distinct Gene and MicroRNA Expression Signatures. J. Clin. Oncol..

[59] Wu C.-Y., Li L., Chen S.-L., Yang X., Zhang C.Z., Cao Y. (2021). A Zic2/Runx2/NOLC1 Signaling Axis Mediates Tumor Growth and Metastasis in Clear Cell Renal Cell Carcinoma. Cell Death Dis..

[60] Wang C., Shi Z., Zhang Y., Li M., Zhu J., Huang Z., Zhang J., Chen J. (2021). CBFβ Promotes Colorectal Cancer Progression through Transcriptionally Activating OPN, FAM129A, and UPP1 in a RUNX2-Dependent Manner. Cell Death Differ..

[61] Liu B., Pan S., Liu J., Kong C. (2021). Cancer-Associated Fibroblasts and the Related Runt-Related Transcription Factor 2 (RUNX2) Promote Bladder Cancer Progression. Gene.

[62] Yang D.-P., Huang W.-Y., Chen G., Chen S.-W., Yang J., He R.-Q., Huang S.-N., Gan T.-Q., Ma J., Yang L.-J. (2020). Clinical Significance of Transcription Factor RUNX2 in Lung Adenocarcinoma and Its Latent Transcriptional Regulating Mechanism. Comput. Biol. Chem..

[63] Ito K., Chuang L.S.H., Ito T., Chang T.L., Fukamachi H., Salto–Tellez M., Ito Y. (2011). Loss of Runx3 Is a Key Event in Inducing Precancerous State of the Stomach. Gastroenterology.

[64] Xiao W.-H., Liu W.-W. (2004). Hemizygous Deletion and Hypermethylation of RUNX3 Gene in Hepatocellular Carcinoma. World J. Gastroenterol..

[65] Zaret K.S., Carroll J.S. (2011). Pioneer Transcription Factors: Establishing Competence for Gene Expression. Genes Dev..

[66] Lichtinger M., Ingram R., Hannah R., Müller D., Clarke D., Assi S.A., Lie-A-Ling M., Noailles L., Vijayabaskar M.S., Wu M. (2012). RUNX1 Reshapes the Epigenetic Landscape at the Onset of Haematopoiesis: RUNX1 Shifts Transcription Factor Binding Patterns. EMBO J..

[67] Hoogenkamp M., Lichtinger M., Krysinska H., Lancrin C., Clarke D., Williamson A., Mazzarella L., Ingram R., Jorgensen H., Fisher A. (2009). Early Chromatin Unfolding by RUNX1: A Molecular Explanation for Differential Requirements during Specification versus Maintenance of the Hematopoietic Gene Expression Program. Blood.

[68] Kitabayashi I. (2001). Activation of AML1-Mediated Transcription by MOZ and Inhibition by the MOZ-CBP Fusion Protein. EMBO J..

[69] Huang G., Zhao X., Wang L., Elf S., Xu H., Zhao X., Sashida G., Zhang Y., Liu Y., Lee J. (2011). The Ability of MLL to Bind RUNX1 and Methylate H3K4 at PU.1 Regulatory Regions Is Impaired by MDS/AML-Associated RUNX1/AML1 Mutations. Blood.

[70] Westendorf J.J., Zaidi S.K., Cascino J.E., Kahler R., Van Wijnen A.J., Lian J.B., Yoshida M., Stein G.S., Li X. (2002). Runx2 (Cbfa1, AML-3) Interacts with Histone Deacetylase 6 and Represses the P21*^CIP1/WAF1^* Promoter. Mol. Cell. Biol..

[71] Yi H., Li G., Long Y., Liang W., Cui H., Zhang B., Tan Y., Li Y., Shen L., Deng D. (2020). Integrative Multi-Omics Analysis of a Colon Cancer Cell Line with Heterogeneous Wnt Activity Revealed RUNX2 as an Epigenetic Regulator of EMT. Oncogene.

[72] Lee J.-W., Kim D.-M., Jang J.-W., Park T.-G., Song S.-H., Lee Y.-S., Chi X.-Z., Park I.Y., Hyun J.-W., Ito Y. (2019). RUNX3 Regulates Cell Cycle-Dependent Chromatin Dynamics by Functioning as a Pioneer Factor of the Restriction-Point. Nat. Commun..

[73] National Library of Medicine RUNX1 RUNX Family Transcription Factor 1 [Homo sapiens (Human)]. https://www.ncbi.nlm.nih.gov/gene/861.

[74] Miyoshi H., Shimizu K., Kozu T., Maseki N., Kaneko Y., Ohki M. (1991). T(8;21) Breakpoints on Chromosome 21 in Acute Myeloid Leukemia Are Clustered within a Limited Region of a Single Gene, AML1. Proc. Natl. Acad. Sci. USA.

[75] Martinez M., Hinojosa M., Trombly D., Morin V., Stein J., Stein G., Javed A., Gutierrez S.E. (2016). Transcriptional Auto-Regulation of RUNX1 P1 Promoter. PLoS ONE.

[76] Lin T.-C. (2022). RUNX1 and Cancer. Biochim. Biophys. Acta (BBA) Rev. Cancer.

[77] TIMER2.0. http://timer.cistrome.org/.

[78] GENT2. http://gent2.appex.kr/gent2/.

[79] Zhu J., Sanborn J.Z., Benz S., Szeto C., Hsu F., Kuhn R.M., Karolchik D., Archie J., Lenburg M.E., Esserman L.J. (2009). The UCSC Cancer Genomics Browser. Nat. Methods.

[80] UCSC Xena. http://xena.ucsc.edu/welcome-to-ucsc-xena/.

[81] Miyagawa K., Sakakura C., Nakashima S., Yoshikawa T., Kin S., Nakase Y., Ito K., Yamagishi H., Ida H., Yazumi S. (2006). Down-Regulation of RUNX1, RUNX3 and CBFbeta in Hepatocellular Carcinomas in an Early Stage of Hepatocarcinogenesis. Anticancer Res..

[82] Wang T., Jin H., Hu J., Li X., Ruan H., Xu H., Wei L., Dong W., Teng F., Gu J. (2020). COL4A1 Promotes the Growth and Metastasis of Hepatocellular Carcinoma Cells by Activating FAK-Src Signaling. J. Exp. Clin. Cancer Res..

[83] Takakura N., Watanabe T., Suenobu S., Yamada Y., Noda T., Ito Y., Satake M., Suda T. (2000). A Role for Hematopoietic Stem Cells in Promoting Angiogenesis. Cell.

[84] Iwatsuki K., Tanaka K., Kaneko T., Kazama R., Okamoto S., Nakayama Y., Ito Y., Satake M., Takahashi S.-I., Miyajima A. (2005). Runx1 Promotes Angiogenesis by Downregulation of Insulin-like Growth Factor-Binding Protein-3. Oncogene.

[85] Ter Elst A., Ma B., Scherpen F.J.G., De Jonge H.J.M., Douwes J., Wierenga A.T.J., Schuringa J.J., Kamps W.A., De Bont E.S.J.M. (2011). Repression of Vascular Endothelial Growth Factor Expression by the Runt-Related Transcription Factor 1 in Acute Myeloid Leukemia. Cancer Res..

[86] Tuo Z., Zhang Y., Wang X., Dai S., Liu K., Xia D., Wang J., Bi L. (2022). RUNX1 Is a Promising Prognostic Biomarker and Related to Immune Infiltrates of Cancer-Associated Fibroblasts in Human Cancers. BMC Cancer.

[87] National Library of Medicine RUNX1-IT1 RUNX1 Intronic Transcript 1 [Homo sapiens (Human)]. https://www.ncbi.nlm.nih.gov/gene?Db=gene&Cmd=DetailsSearch&Term=80215.

[88] Yan P.-H., Wang L., Chen H., Yu F.-Q., Guo L., Liu Y., Zhang W.-J., Bai Y.-L. (2019). LncRNA RUNX1-IT1 Inhibits Proliferation and Promotes Apoptosis of Hepatocellular Carcinoma by Regulating MAPK Pathways. Eur. Rev. Med. Pharmacol. Sci..

[89] Sun L., Wang L., Chen T., Shi Y., Yao B., Liu Z., Wang Y., Li Q., Liu R., Niu Y. (2020). LncRNA RUNX1-IT1 Which Is Downregulated by Hypoxia-Driven Histone Deacetylase 3 Represses Proliferation and Cancer Stem-like Properties in Hepatocellular Carcinoma Cells. Cell Death Dis..

[90] Vivacqua A., De Marco P., Santolla M.F., Cirillo F., Pellegrino M., Panno M.L., Abonante S., Maggiolini M. (2015). Estrogenic Gper Signaling Regulates Mir144 Expression in Cancer Cells and Cancer-Associated Fibroblasts (Cafs). Oncotarget.

[91] Qin L., Zhang Y., Lin J., Shentu Y., Xie X. (2016). MicroRNA-455 Regulates Migration and Invasion of Human Hepatocellular Carcinoma by Targeting Runx2. Oncol. Rep..

[92] Wang S.-Y., Chen C.-L., Hu Y.-C., Chi Y., Huang Y.-H., Su C.-W., Jeng W.-J., Liang Y.-J., Wu J.-C. (2019). High Expression of MicroRNA-196a Is Associated with Progression of Hepatocellular Carcinoma in Younger Patients. Cancers.

[93] Wang L., Feng Y., Zhang C., Chen X., Huang H., Li W., Zhang J., Liu Y. (2021). Upregulation of OGT by Caveolin-1 Promotes Hepatocellular Carcinoma Cell Migration and Invasion. Cell Biol. Int..

[94] Huang H., Liu Y., Yu P., Qu J., Guo Y., Li W., Wang S., Zhang J. (2018). MiR-23a Transcriptional Activated by Runx2 Increases Metastatic Potential of Mouse Hepatoma Cell via Directly Targeting Mgat3. Sci. Rep..

[95] Jing G., Zheng X., Ji X. (2021). LncRNA HAND2-AS1 Overexpression Inhibits Cancer Cell Proliferation in Hepatocellular Carcinoma by Downregulating RUNX2 Expression. J. Clin. Lab. Anal..

[96] Yu W., Qiao Y., Tang X., Ma L., Wang Y., Zhang X., Weng W., Pan Q., Yu Y., Sun F. (2014). Tumor Suppressor Long Non-Coding RNA, MT1DP Is Negatively Regulated by YAP and Runx2 to Inhibit FoxA1 in Liver Cancer Cells. Cell. Signal..

[97] Chen Z., Zuo X., Pu L., Zhang Y., Han G., Zhang L., Wu J., Wang X. (2019). Circ LARP 4 Induces Cellular Senescence through Regulating MiR-761/RUNX 3/P53/P21 Signaling in Hepatocellular Carcinoma. Cancer Sci..

[98] Gou Y., Zhai F., Zhang L., Cui L. (2017). RUNX3 Regulates Hepatocellular Carcinoma Cell Metastasis via Targeting MiR-186/E-Cadherin/EMT Pathway. Oncotarget.

[99] Li S., Li F., Xu L., Liu X., Zhu X., Gao W., Shen X. (2022). TLR2 Agonist Promotes Myeloid-Derived Suppressor Cell Polarization via Runx1 in Hepatocellular Carcinoma. Int. Immunopharmacol..

[100] Hill T.P., Später D., Taketo M.M., Birchmeier W., Hartmann C. (2005). Canonical Wnt/β-Catenin Signaling Prevents Osteoblasts from Differentiating into Chondrocytes. Dev. Cell.

[101] Zhang Y.-W., Yasui N., Ito K., Huang G., Fujii M., Hanai J., Nogami H., Ochi T., Miyazono K., Ito Y. (2000). A *RUNX2/PEBP2* AA/*CBFA1* Mutation Displaying Impaired Transactivation and Smad Interaction in Cleidocranial Dysplasia. Proc. Natl. Acad. Sci. USA.

[102] Zhao W., Yang H., Chai J., Xing L. (2021). RUNX2 as a Promising Therapeutic Target for Malignant Tumors. Cancer Manag. Res..

[103] Wang Q., Yu W., Huang T., Zhu Y., Huang C. (2016). RUNX2 Promotes Hepatocellular Carcinoma Cell Migration and Invasion by Upregulating MMP9 Expression. Oncol. Rep..

[104] Cao Z., Sun B., Zhao X., Zhang Y., Gu Q., Liang X., Dong X., Zhao N. (2020). Correction: Cao, Z.; et al. The Expression and Functional Significance of Runx2 in Hepatocellular Carcinoma: Its Role in Vasculogenic Mimicry and Epithelial—Mesenchymal Transition. Int. J. Mol. Sci..

[105] Emma M.R., Iovanna J.L., Bachvarov D., Puleio R., Loria G.R., Augello G., Candido S., Libra M., Gulino A., Cancila V. (2016). NUPR1, a New Target in Liver Cancer: Implication in Controlling Cell Growth, Migration, Invasion and Sorafenib Resistance. Cell Death Dis..

[106] Wang X., Li L., Wu Y., Zhang R., Zhang M., Liao D., Wang G., Qin G., Xu R., Kang T. (2016). CBX4 Suppresses Metastasis via Recruitment of HDAC3 to the Runx2 Promoter in Colorectal Carcinoma. Cancer Res..

[107] Sase T., Suzuki T., Miura K., Shiiba K., Sato I., Nakamura Y., Takagi K., Onodera Y., Miki Y., Watanabe M. (2012). Runt-Related Transcription Factor 2 in Human Colon Carcinoma: A Potent Prognostic Factor Associated with Estrogen Receptor. Int. J. Cancer.

[108] Komori T. (2002). Runx2, A Multifunctional Transcription Factor in Skeletal Development. J. Cell. Biochem..

[109] Pratap J., Javed A., Languino L.R., Van Wijnen A.J., Stein J.L., Stein G.S., Lian J.B. (2005). The Runx2 Osteogenic Transcription Factor Regulates Matrix Metalloproteinase 9 in Bone Metastatic Cancer Cells and Controls Cell Invasion. Mol. Cell. Biol..

[110] Boregowda R.K., Olabisi O.O., Abushahba W., Jeong B.-S., Haenssen K.K., Chen W., Chekmareva M., Lasfar A., Foran D.J., Goydos J.S. (2014). RUNX2 Is Overexpressed in Melanoma Cells and Mediates Their Migration and Invasion. Cancer Lett..

[111] Li X.-Q., Du X., Li D.-M., Kong P.-Z., Sun Y., Liu P.-F., Wang Q.-S., Feng Y.-M. (2015). ITGBL1 Is a Runx2 Transcriptional Target and Promotes Breast Cancer Bone Metastasis by Activating the TGFβ Signaling Pathway. Cancer Res..

[112] El-Gendi S.M., Mostafa M.F. (2016). Runx2 Expression as a Potential Prognostic Marker in Invasive Ductal Breast Carcinoma. Pathol. Oncol. Res..

[113] Baniwal S.K., Khalid O., Gabet Y., Shah R.R., Purcell D.J., Mav D., Kohn-Gabet A.E., Shi Y., Coetzee G.A., Frenkel B. (2010). Runx2 Transcriptome of Prostate Cancer Cells: Insights into Invasiveness and Bone Metastasis. Mol. Cancer.

[114] Meng J., Sun B., Zhao X., Zhang D., Zhao X., Gu Q., Dong X., Zhao N., Liu P., Liu Y. (2014). Doxycycline as an Inhibitor of the Epithelial-to-Mesenchymal Transition and Vasculogenic Mimicry in Hepatocellular Carcinoma. Mol. Cancer Ther..

[115] Sun T., Zhao N., Zhao X., Gu Q., Zhang S., Che N., Wang X., Du J., Liu Y., Sun B. (2010). Expression and Functional Significance of Twist1 in Hepatocellular Carcinoma: Its Role in Vasculogenic Mimicry. Hepatology.

[116] Funasaka T., Raz A., Nangia-Makker P. (2014). Galectin-3 in Angiogenesis and Metastasis. Glycobiology.

[117] Vestweber D. (2008). VE-Cadherin: The Major Endothelial Adhesion Molecule Controlling Cellular Junctions and Blood Vessel Formation. Arterioscler. Thromb. Vasc. Biol..

[118] Yang N., Wang L., Chen T., Liu R., Liu Z., Zhang L. (2020). ZNF521 Which Is Downregulated by MiR-802 Suppresses Malignant Progression of Hepatocellular Carcinoma through Regulating Runx2 Expression. J. Cancer.

[119] Li Y.-J., Ping C., Tang J., Zhang W. (2016). MicroRNA-455 Suppresses Non-Small Cell Lung Cancer through Targeting ZEB1: The Role of MiRNA-455 in NSCLC. Cell Biol. Int..

[120] Chai J., Wang S., Han D., Dong W., Xie C., Guo H. (2015). MicroRNA-455 Inhibits Proliferation and Invasion of Colorectal Cancer by Targeting RAF Proto-Oncogene Serine/Threonine-Protein Kinase. Tumor Biol..

[121] Chou C.-H., Shrestha S., Yang C.-D., Chang N.-W., Lin Y.-L., Liao K.-W., Huang W.-C., Sun T.-H., Tu S.-J., Lee W.-H. (2018). MiRTarBase Update 2018: A Resource for Experimentally Validated MicroRNA-Target Interactions. Nucleic Acids Res..

[122] Zhao W., Zhang S., Wang B., Huang J., Lu W.W., Chen D. (2016). Runx2 and MicroRNA Regulation in Bone and Cartilage Diseases: Runx2 and MiRNAs in Bone and Cartilage. Ann. N. Y. Acad. Sci..

[123] Wai P.Y., Kuo P.C. (2004). The Role of Osteopontin in Tumor Metastasis. J. Surg. Res..

[124] Whittle M.C., Izeradjene K., Rani P.G., Feng L., Carlson M.A., DelGiorno K.E., Wood L.D., Goggins M., Hruban R.H., Chang A.E. (2015). RUNX3 Controls a Metastatic Switch in Pancreatic Ductal Adenocarcinoma. Cell.

[125] Kumar A., Sundaram S., Rayala S.K., Venkatraman G. (2017). UnPAKing RUNX3 Functions–Both Sides of the Coin. Small GTPases.

[126] Zhang X., He H., Zhang X., Guo W., Wang Y. (2015). RUNX3 Promoter Methylation Is Associated with Hepatocellular Carcinoma Risk: A Meta-Analysis. Cancer Investig..

[127] Yang Y., Ye Z., Zou Z., Xiao G., Luo G., Yang H. (2014). Clinicopathological Significance of RUNX3 Gene Hypermethylation in Hepatocellular Carcinoma. Tumor Biol..

[128] El-shaarawy F., Abo ElAzm M.M., Mohamed R.H., Radwan M.I., Abo-Elmatty D.M., Mehanna E.T. (2022). Relation of the Methylation State of RUNX3 and P16 Gene Promoters with Hepatocellular Carcinoma in Egyptian Patients. Egypt. J. Med. Hum. Genet..

[129] Sun G., Zhang C., Feng M., Liu W., Xie H., Qin Q., Zhao E., Wan L. (2017). Methylation Analysis of P16, SLIT2, SCARA5, and Runx3 Genes in Hepatocellular Carcinoma. Medicine.

[130] El-Bendary M., Nour D., Arafa M., Neamatallah M. (2020). Methylation of Tumour Suppressor Genes *RUNX3, RASSF1A* and *E-Cadherin* in HCV-Related Liver Cirrhosis and Hepatocellular Carcinoma. Br. J. Biomed. Sci..

[131] Chen F., Liu X., Bai J., Pei D., Zheng J. (2016). The Emerging Role of RUNX3 in Cancer Metastasis (Review). Oncol. Rep..

[132] Gao J., Chen Y., Wu K.-C., Liu J., Zhao Y.-Q., Pan Y.-L., Du R., Zheng G.-R., Xiong Y.-M., Xu H.-L. (2010). RUNX3 Directly Interacts with Intracellular Domain of Notch1 and Suppresses Notch Signaling in Hepatocellular Carcinoma Cells. Exp. Cell Res..

[133] Chi X.-Z., Lee J.-W., Lee Y.-S., Park I.Y., Ito Y., Bae S.-C. (2017). Runx3 Plays a Critical Role in Restriction-Point and Defense against Cellular Transformation. Oncogene.

[134] Shiraha H., Nishina S., Yamamoto K. (2011). Loss of Runt-Related Transcription Factor 3 Causes Development and Progression of Hepatocellular Carcinoma. J. Cell. Biochem..

[135] Tanaka S., Shiraha H., Nakanishi Y., Nishina S.-I., Matsubara M., Horiguchi S., Takaoka N., Iwamuro M., Kataoka J., Kuwaki K. (2012). Runt-Related Transcription Factor 3 Reverses Epithelial-Mesenchymal Transition in Hepatocellular Carcinoma. Int. J. Cancer.

[136] Kataoka J., Shiraha H., Horiguchi S., Sawahara H., Uchida D., Nagahara T., Iwamuro M., Morimoto H., Takeuchi Y., Kuwaki K. (2016). Loss of Runt-Related Transcription Factor 3 Induces Resistance to 5-Fluorouracil and Cisplatin in Hepatocellular Carcinoma. Oncol. Rep..

[137] Xu N., Shen C., Luo Y., Xia L., Xue F., Xia Q., Zhang J. (2012). Upregulated MiR-130a Increases Drug Resistance by Regulating RUNX3 and Wnt Signaling in Cisplatin-Treated HCC Cell. Biochem. Biophys. Res. Commun..

[138] Chen W., Gao N., Shen Y., Cen J. (2010). Hypermethylation Downregulates Runx3 Gene Expression and Its Restoration Suppresses Gastric Epithelial Cell Growth by Inducing P27 and Caspase3 in Human Gastric Cancer. J. Gastroenterol. Hepatol..

[139] Wei D., Gong W., Oh S.C., Li Q., Kim W.D., Wang L., Le X., Yao J., Wu T.T., Huang S. (2005). Loss of RUNX3 Expression Significantly Affects the Clinical Outcome of Gastric Cancer Patients and Its Restoration Causes Drastic Suppression of Tumor Growth and Metastasis. Cancer Res..

[140] Chi X.-Z., Yang J.-O., Lee K.-Y., Ito K., Sakakura C., Li Q.-L., Kim H.-R., Cha E.-J., Lee Y.-H., Kaneda A. (2005). RUNX3 Suppresses Gastric Epithelial Cell Growth by Inducing *P21^WAF1^*^/*Cip1*^ Expression in Cooperation with Transforming Growth Factor β-Activated SMAD. Mol. Cell. Biol..

[141] Ito Y., Miyazono K. (2003). RUNX Transcription Factors as Key Targets of TGF-β Superfamily Signaling. Curr. Opin. Genet. Dev..

[142] Yano T., Ito K., Fukamachi H., Chi X.-Z., Wee H.-J., Inoue K., Ida H., Bouillet P., Strasser A., Bae S.-C. (2006). The RUNX3 Tumor Suppressor Upregulates Bim in Gastric Epithelial Cells Undergoing Transforming Growth Factorβ-Induced Apoptosis. Mol. Cell. Biol..

[143] Bae S.-C., Choi J.-K. (2004). Tumor Suppressor Activity of RUNX3. Oncogene.

[144] Zaidi S.K., Sullivan A.J., Van Wijnen A.J., Stein J.L., Stein G.S., Lian J.B. (2002). Integration of Runx and Smad Regulatory Signals at Transcriptionally Active Subnuclear Sites. Proc. Natl. Acad. Sci. USA.

[145] Zeng S., Shen W., Liu L. (2018). Senescence and Cancer. Cancer Transl. Med..

[146] Sweeney K., Cameron E.R., Blyth K. (2020). Complex Interplay between the RUNX Transcription Factors and Wnt/β-Catenin Pathway in Cancer: A Tango in the Night. Mol. Cells.

[147] Zhang Y., Wei W., Cheng N., Wang K., Li B., Jiang X., Sun S. (2012). Hepatitis C Virus-Induced up-Regulation of MicroRNA-155 Promotes Hepatocarcinogenesis by Activating Wnt Signaling. Hepatology.

[148] Giovannini C., Fornari F., Piscaglia F., Gramantieri L. (2021). Notch Signaling Regulation in HCC: From Hepatitis Virus to Non-Coding RNAs. Cells.

[149] Ahn S., Hyeon J., Park C.-K. (2013). Notchl and Notch4 Are Markers for Poor Prognosis of Hepatocellular Carcinoma. Hepatobiliary Pancreat. Dis. Int..

[150] Nishina S.-I. (2011). Restored Expression of the Tumor Suppressor Gene RUNX3 Reduces Cancer Stem Cells in Hepatocellular Carcinoma by Suppressing Jagged1-Notch Signaling. Oncol. Rep..

[151] Chai M.Y., Kou B.X., Fu Z., Wei F.L., Dou S.S., Chen D.X., Liu X.N. (2022). Sorafenib regulates vascular endothelial growth factor by runt-related transcription factor-3 to inhibit angiogenesis in hepatocellular carcinoma. Zhonghua Gan Zang Bing Za Zhi.

[152] Lee S.H., Bae S.C., Kim K.W., Lee Y.M. (2014). RUNX3 Inhibits Hypoxia-Inducible Factor-1α Protein Stability by Interacting with Prolyl Hydroxylases in Gastric Cancer Cells. Oncogene.

[153] Galle P.R., Forner A., Llovet J.M., Mazzaferro V., Piscaglia F., Raoul J.-L., Schirmacher P., Vilgrain V. (2018). EASL Clinical Practice Guidelines: Management of Hepatocellular Carcinoma. J. Hepatol..

[154] Lamouille S., Xu J., Derynck R. (2014). Molecular Mechanisms of Epithelial–Mesenchymal Transition. Nat. Rev. Mol. Cell Biol..

[155] Voon D.C.-C., Wang H., Koo J.K.W., Nguyen T.A.P., Hor Y.T., Chu Y.-S., Ito K., Fukamachi H., Chan S.L., Thiery J.P. (2012). Runx3 Protects Gastric Epithelial Cells Against Epithelial-Mesenchymal Transition-Induced Cellular Plasticity and Tumorigenicity. Stem Cells.

[156] Loh C.-Y., Chai J., Tang T., Wong W., Sethi G., Shanmugam M., Chong P., Looi C. (2019). The E-Cadherin and N-Cadherin Switch in Epithelial-to-Mesenchymal Transition: Signaling, Therapeutic Implications, and Challenges. Cells.

[157] Lee S.H., Jung Y.D., Choi Y.S., Lee Y.M. (2015). Targeting of RUNX3 by MiR-130a and MiR-495 Cooperatively Increases Cell Proliferation and Tumor Angiogenesis in Gastric Cancer Cells. Oncotarget.

[158] Wang M., Wang X., Liu W. (2020). MicroRNA-130a-3p Promotes the Proliferation and Inhibits the Apoptosis of Cervical Cancer Cells via Negative Regulation of RUNX3. Mol. Med. Rep..

[159] Tang L., Chen R., Xu X. (2020). Synthetic Lethality: A Promising Therapeutic Strategy for Hepatocellular Carcinoma. Cancer Lett..

[160] Llovet J.M., Ricci S., Mazzaferro V., Hilgard P., Gane E., Blanc J.-F., De Oliveira A.C., Santoro A., Raoul J.-L., Forner A. (2008). Sorafenib in Advanced Hepatocellular Carcinoma. N. Engl. J. Med..

[161] Boyault S., Rickman D.S., De Reyniès A., Balabaud C., Rebouissou S., Jeannot E., Hérault A., Saric J., Belghiti J., Franco D. (2007). Transcriptome Classification of HCC Is Related to Gene Alterations and to New Therapeutic Targets. Hepatology.

[162] Schulze K., Nault J.-C., Villanueva A. (2016). Genetic Profiling of Hepatocellular Carcinoma Using Next-Generation Sequencing. J. Hepatol..

[163] Jhunjhunwala S., Jiang Z., Stawiski E.W., Gnad F., Liu J., Mayba O., Du P., Diao J., Johnson S., Wong K.-F. (2014). Diverse Modes of Genomic Alteration in Hepatocellular Carcinoma. Genome Biol..

[164] Ally A., Balasundaram M., Carlsen R., Chuah E., Clarke A., Dhalla N., Holt R.A., Jones S.J.M., Lee D., Ma Y. (2017). Comprehensive and Integrative Genomic Characterization of Hepatocellular Carcinoma. Cell.

[165] Yang C., Guo Y., Qian R., Huang Y., Zhang L., Wang J., Huang X., Liu Z., Qin W., Wang C. (2021). Mapping the Landscape of Synthetic Lethal Interactions in Liver Cancer. Theranostics.

[166] Bae S.-C., Kolinjivadi A.M., Ito Y. (2019). Functional Relationship between P53 and RUNX Proteins. J. Mol. Cell Biol..

[167] Krishnan V. (2023). The RUNX Family of Proteins, DNA Repair, and Cancer. Cells.

[168] Pharos. https://pharos.nih.gov/targets/RUNX2.

[169] Lu X.X., Zhu L.Q., Pang F., Sun W., Ou C., Li Y., Cao J., Hu Y.L. (2014). Relationship between RUNX3 Methylation and Hepatocellular Carcinoma in Asian Populations: A Systematic Review. Genet. Mol. Res..

[170] Jovanovic-Cupic S., Bozovic A., Krajnovic M., Petrovic N., Shahid I. (2018). Hepatitis C: Host and Viral Factors Associated with Response to Therapy and Progression of Liver Fibrosis. Hepatitis C—From Infection to Cure.

